# Controlled release of liraglutide using thermogelling polymers in treatment of diabetes

**DOI:** 10.1038/srep31593

**Published:** 2016-08-17

**Authors:** Yipei Chen, Yuzhuo Li, Wenjia Shen, Kun Li, Lin Yu, Qinghua Chen, Jiandong Ding

**Affiliations:** 1 State Key Laboratory of Molecular Engineering of Polymers, Collaborative Innovation Center of Polymers and Polymer Composite Materials, Department of Macromolecular Science, Fudan University, Shanghai, 200433, China; 2 National Pharmaceutical Engineering Research Center, China State Institute of Pharmaceutical Industry, Shanghai, 200437, China

## Abstract

In treatment of diabetes, it is much desired in clinics and challenging in pharmaceutics and material science to set up a long-acting drug delivery system. This study was aimed at constructing a new delivery system using thermogelling PEG/polyester copolymers. Liraglutide, a fatty acid-modified antidiabetic polypeptide, was selected as the model drug. The thermogelling polymers were presented by poly(*ε*-caprolactone-*co*-glycolic acid)-poly(ethylene glycol)-poly(*ε*-caprolactone-*co*-glycolic acid) (PCGA-PEG-PCGA) and poly(lactic acid-*co*-glycolic acid)-poly(ethylene glycol)-poly(lactic acid-*co*-glycolic acid) (PLGA-PEG-PLGA). Both the copolymers were soluble in water, and their concentrated solutions underwent temperature-induced sol-gel transitions. The drug-loaded polymer solutions were injectable at room temperature and gelled *in situ* at body temperature. Particularly, the liraglutide-loaded PCGA-PEG-PCGA thermogel formulation exhibited a sustained drug release manner over one week in both *in vitro* and *in vivo* tests. This feature was attributed to the combined effects of an appropriate drug/polymer interaction and a high chain mobility of the carrier polymer, which facilitated the sustained diffusion of drug out of the thermogel. Finally, a single subcutaneous injection of this formulation showed a remarkably improved glucose tolerance of mice for one week. Hence, the present study not only developed a promising long-acting antidiabetic formulation, but also put forward a combined strategy for controlled delivery of polypeptide.

Type 2 diabetes mellitus (T2DM) is a global epidemic characterized by hyperglycemia. With the increase of patient number at an alarming rate, T2DM and its complications have got to be one of the most threatening and challenging problems concerning public health around the world, and the consequent health, social and economic burden is great. Recently, incretin-based therapies have been acknowledged as an effective treatment strategy for patients with T2DM^1^,^2^,^3^. Glucagon-like peptide-1 (GLP-1), a hormone of 31 amino acid residues, is released from the L cells in the colon and the ileum, and stimulates insulin secretion in a glucose-dependent manner. Therefore, GLP-1 effectively controls the blood glucose level of T2DM patients without the risk of hypoglycemia^1^,^4^. However, the clinical application of GLP-1 is limited by its short half-life (about 2 min) due to the rapid degradation by dipeptidyl peptidase IV (DPP-IV). To resolve this problem, DPP-IV-resistant GLP-1 analogues have been developed^1^,^2^.

Liraglutide (Lira) is a fatty acid derivative of GLP-1 and its amino acid sequence is displayed in Fig. 1a. It is formed by attaching a 16-carbon fatty acid molecule at position Lys26 and making an Arg34Lys substitution on GLP-1, and shares approximately 97% sequence homology with GLP-1^5^,^6^. These structural modifications of Lira increase chain aggregation, promote reversible non-covalent binding to other molecules, such as albumin, and resist DPP-IV degradation. Thus, the half-life of Lira is prolonged to about 13 h after subcutaneous (SC) injection into human body^6^. Meanwhile, Lira retains the physiological activities of GLP-1, which include stimulating insulin release and suppressing glucagon secretion in a glucose-dependent manner, improving *β*-cell mass and function, decreasing insulin resistance, delaying gastric emptying, and increasing satiety^7^,^8^,^9^.

Victoza, the solution injection of Lira developed by Novo Nordisk Inc., has been approved by both the European Medicine Agency (2009) and the U.S. Food and Drug Administration (FDA) (2010) for the treatment of T2DM. As a blockbuster in the antidiabetic drug market, the global sales of Victoza reached $420 million for the year (2010) and quickly increased to $2700 million in 2015. In general, once-daily SC injection of Victoza is needed for patients with T2DM. The dosage usually begins with 0.6 mg, and then increases to 1.2 mg after one week. It is obvious that the repeated administration and complex treatment regimen is not convenient for patients, and thus a long-acting drug delivery system of Lira is much desired in order to reduce administration frequency and improve patients’ compliance. However, no long-acting formulation of Lira has, to the best of our knowledge, been reported.

As minimally invasive depot forming materials, thermogelling polymers have been extensively studied in the past decade^10^,^11^,^12^,^13^,^14^,^15^,^16^,^17^. They are dissolved in water at low or room temperature and exhibit a sol-gel transition with increase of temperature. The drugs or cells can be easily incorporated into thermogels by simply mixing them with polymeric aqueous solutions at low temperatures. This procedure avoids the denaturation of sensitive therapeutic agents, such as protein or polypeptide, due to free of any organic solvent and high temperature. Once administrated by a conventional syringe, the polymer aqueous solution spontaneously transforms into a physical hydrogel at the injection site. This *in situ* forming gel containing drugs or cells can act as a sustained drug release depot or a cell-growing matrix^12^,^18^,^19^,^20^,^21^,^22^,^23^,^24^. Generally, the delicate balance between hydrophilicity and hydrophobicity of an amphiphilic polymer plays a crucial role in the thermo-induced physical gelation^25^,^26^,^27^. To date, polyester^27^,^28^, polypeptide^19^,^29^,^30^, poly(phosphazenes)^31^,^32^, and so on have been employed as biodegradable hydrophobic segments, while PEG has been utilized as a hydrophilic block.

Among the polymers undergoing a thermo-reversible sol-gel transition in water, block copolymers composed of hydrophobic polyesters, such as poly(lactic acid-*co*-glycolic acid) (PLGA), poly(*ε*-caprolactone) (PCL), poly (*ε*-caprolactone-*co*-glycolic acid) (PCGA), and hydrophilic PEG are particularly interesting and important because of the good safety profile and facile synthesis^33^,^34^,^35^. Their thermogelling properties and degradation behavior can be well-modulated by many molecular parameters including molecular weight (MW), MW distribution, polyester composition, block ratio, sequence of polyester block, as well as end group^25^,^26^,^28^,^35^,^36^,^37^,^38^. The corresponding mechanism of thermogelation is attributed to the formation of a percolated micelle network as the temperature increases^27^,^39^, as illustrated in Fig. 1b. These resulting thermogels have been suggested as promising biomaterials for delivery of a variety of drugs^40^,^41^,^42^, and other biomedical applications^18^,^43^,^44^,^45^,^46^,^47^. Generally, the release profiles of drugs from thermogels can be greatly affected by many factors, such as polymer MW, polymer concentration, drug loading amount and even additive excipient^41^,^48^,^49^,^50^. Nevertheless, little attention has being paid to the effect of PEG/polyester thermogels with different polyester components on the release behavior of drugs. In fact, various polyester segments differ in their hydrophobicity, chain mobility, degradation rate, permeability to drugs, and so on^51^,^52^,^53^,^54^.

In this study, a long-acting delivery system of Lira in treatment of T2DM using thermogelling block copolymers as the carriers was examined for the first time. Different from hydrophilic polypeptides such as exenatide and insulin, Lira is of relatively higher global hydrophobicity due to the introduction of 16-carbon fatty acid into the peptide backbone, resulting in the increased capacity of chain aggregation and non-covalent binding to other amphiphilic molecules^1^,^6^. Consequently, we hypothesized that Lira could interact with amphiphilic PEG/polyester polymers. Their appropriate interactions might realize the release of Lira at a relatively slow and consistent rate, as schematically presented in Fig. 1c.

To verify our hypothesis, two thermogelling triblock copolymers poly(*ε*-caprolactone-*co*-glycolic acid)-poly(ethylene glycol)-poly(*ε*-caprolactone-*co*-glycolic acid) (PCGA-PEG-PCGA) and poly(lactic acid-*co*-glycolic acid)-poly(ethylene glycol)-poly(lactic acid-*co*-glycolic acid) (PLGA-PEG-PLGA) were synthesized. Their physicochemical properties and thermogelling behavior in water were examined. Micellizations of amphiphilic block copolymers with or without the presence of the drug were monitored, and the secondary structures of the polypeptide in the presence of block copolymers were detected. *In vitro* release behavior of Lira from the two thermogels was investigated, and the effect of polyester component on release profile was discussed. Finally, we evaluated the *in vivo* drug release and efficacy of an optimal Lira-loaded thermogel formulation.

## Results

### Characterization of triblock copolymers

Two triblock copolymers PCGA-PEG-PCGA and PLGA-PEG-PLGA were synthesized by us, and characterized via proton nuclear magnetic resonance (^1^H NMR) and gel permeation chromatography (GPC). ^1^H NMR spectra of both the polymers are presented in the Supplementary Fig. S1. The glass transition temperatures (*T*_g_) of the two specimens were also determined by differential scanning calorimetry (DSC). The *T*_g_ of PCGA-PEG-PCGA was −57 °C, which was significantly lower than that of PLGA-PEG-PLGA (−4 °C). This feature indicates that the PCGA-PEG-PCGA system has the higher chain mobility at body temperature compared with PLGA-PEG-PLGA. The detailed physical parameters of the two copolymers used in this study are summarized in Supplementary Table S1.

### Thermogellability of copolymer aqueous solutions

**Figure 2:**
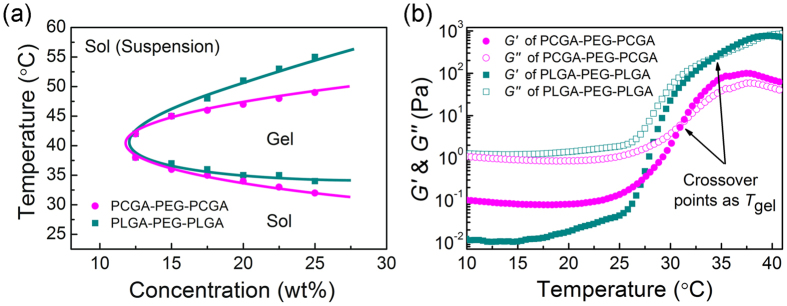
Thermogellability of copolymer aqueous solutions. **(a)** Phase diagrams of PCGA-PEG-PCGA and PLGA-PEG-PLGA triblock copolymers in PBS (pH 7.4). **(b)** Storage modulus *G*′ and loss modulus *G*″ of the indicated samples in PBS (pH 7.4, 25 wt%) as a function of temperature. Heating rates: 0.5 °C/min, oscillatory frequency: 10 rad/s.

Both the PCGA-PEG-PCGA and PLGA-PEG-PLGA polymers were soluble in water at ambient temperature and underwent sol-gel transitions with increasing temperature. Figure 2a presents their phase diagrams in phosphate buffer saline (PBS, pH 7.4), which were determined by the vial inverting method^25^,^35^. The two specimens exhibited a similar critical gel concentration (about 12 wt%). The sol-gel transition temperatures (*T*_gel_) of PCGA-PEG-PCGA and PLGA-PEG-PLGA aqueous solutions with the indicated concentrations were also obtained via the phase diagram. The gel windows of both polymer/water systems covered body temperature, indicating that the two thermogels are suitable for biomedical applications.

The sol-gel transitions of triblock copolymers in PBS were further investigated by dynamic rheological measurements. Figure 2b shows the change in modulus of PCGA-PEG-PCGA and PLGA-PEG-PLGA aqueous solutions (25 wt%) as a function of temperature. At low or room temperature, the moduli were low, and the loss modulus *G*″ was obviously greater than the storage modulus *G*′. This feature reflected their free-flowing sol states. An abrupt increase in modulus was observed along with the formation of *in situ* physical hydrogels as the temperature increased. The crossover point of *G*′ and *G*″ is usually regarded as *T*_gel_^55^. The *T*_gel_ values determined via rheological measurements for the two polymer/water systems (25 wt%) were about 32 °C and 34 °C, which well coincided with those obtained by the vial inverting method. It is noteworthy that the maximum value of *G*′ (*G′*_max_) of PCGA-PEG-PCGA system was one order of magnitude lower than that of PLGA-PEG-PLGA system, which was attributed to the high flexibility of PCGA chains in PCGA-PEG-PCGA polymers.

### Interactions between Lira and PEG/polyester triblock copolymers

**Figure 3:**
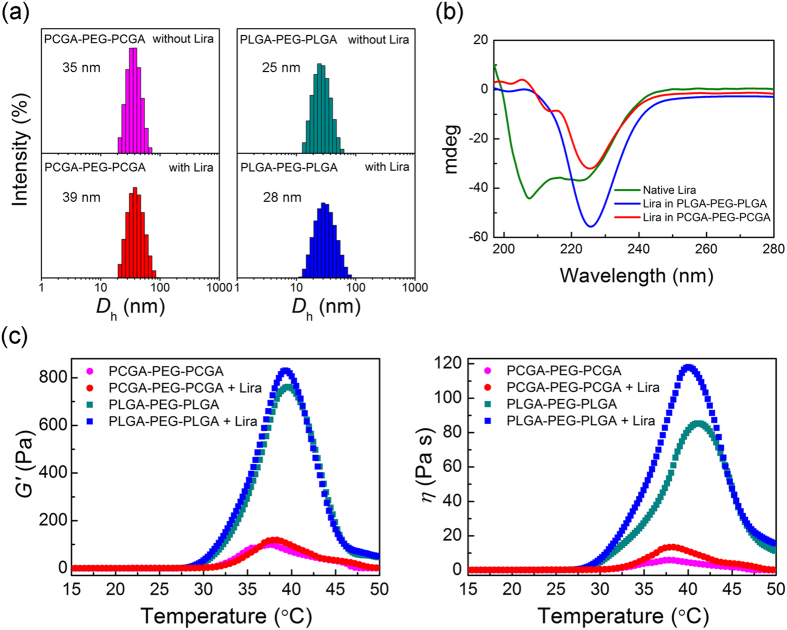
Interactions between Lira and PEG/polyester triblock copolymers. **(a)** Distribution of hydrodynamic diameters of copolymer micelles in water with or without Lira measured by DLS at 25 °C. The copolymer concentration was 0.1 wt% and Lira loading concentration was 0.008 mg/mL. **(b)** CD spectra of native Lira, Lira in PLGA-PEG-PLGA and Lira in PCGA-PEG-PCGA aqueous solutions. Lira concentration was set as 0.04 mg/mL and the polymer concentration was 0.5 wt%. **(c)** Effects of Lira on storage modulus *G*′ and viscosity *η* of the indicated copolymer aqueous solutions (pH 7.4, 25 wt%) at different temperatures. Drug loading concentration: 2 mg/mL, heating rates: 0.5 °C/min, oscillatory frequency: 10 rad/s.

The interactions between Lira and copolymers in water were first studied by dynamic laser scattering (DLS). As shown in Fig. 3a, both the PCGA-PEG-PCGA and PLGA-PEG-PLGA triblock copolymers formed micelles in water with sizes of about 40 nm and 30 nm, respectively. The incorporation of Lira did not influence their unimodal distribution, yet a slightly increase in the micelle size was observed.

The circular dichroism (CD) spectra of Lira were recorded to determine its secondary structure in the different polymer aqueous systems. As shown in Fig. 3b, native Lira showed negative bands with two minima at approximately 208 and 222 nm on the CD spectra, indicating the *α*-helix conformation of Lira in native state. The secondary structure of Lira underwent a significant change in the presence of triblock copolymers. In the case of PCGA-PEG-PCGA, the shifts of negative bands from 208 nm to 213 nm, 222 nm to 225 nm were observed. For the PLGA-PEG-PLGA system, the negative signal at 208 nm disappeared and a bigger shift of negative band from 222 nm to 225 nm was seen. It is obvious that the interactions between the carrier polymers and drug were responsible for the transition of conformation of Lira.

The rheological properties of PEG/polyester copolymers with or without Lira in PBS (pH 7.4) were further investigated, as shown in Fig. 3c. The incorporation of Lira into the polymer aqueous solutions had no obvious influence on their sol-gel transition temperatures. Nevertheless, the presence of Lira caused an increase in both *G*′ and viscosity *η* of the polymer/water systems. It is not difficult to infer that the increases of *G*′ and *η* are also attributed to the interactions between the copolymer chains and polypeptide molecules.

### *In vitro* release of Lira from thermogels

**Figure 4:**
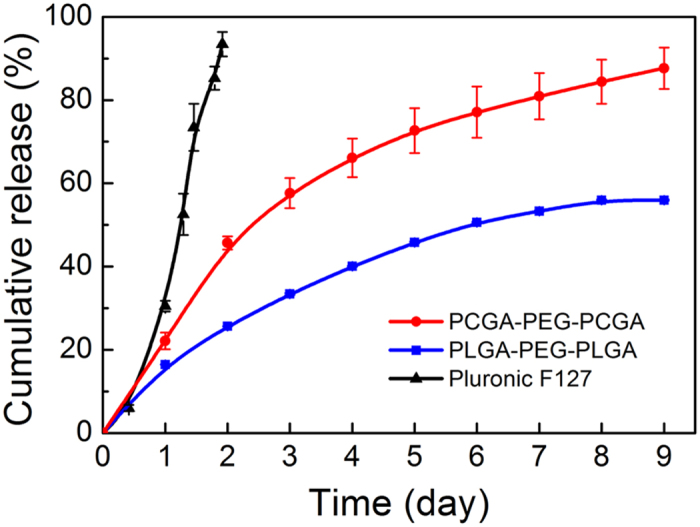
Cumulative release of Lira from the indicated thermogels in PBS (pH 7.4) at 37 °C. The data are represented as the mean ± SD, *n* = 3 for each group. The polymer concentration was 25 wt% and the drug loading amount was 2 mg/mL. The lines are used just for the guides of the eyes.

We evaluated the *in vitro* release profiles of Lira from the thermogel systems. The data in Fig. 4 show the cumulative amounts of released Lira during 9 days. Although the release of Lira from the PLGA-PEG-PLGA hydrogel exhibited a low burst with less than 17% of the loaded amount being released in the first day, a significantly incomplete release was observed at the late stage and only 56% loaded drug was released within 9 days. Different from the PLGA-PEG-PLGA system, the PCGA-PEG-PCGA thermogel formulation showed a sustained release profile over 9 days and the cumulative release amount was more than 85%. This finding indicates that the polyester component played an important role in the sustained drug release from the PEG/polyester thermogel systems.

We also utilized a commercial Pluronic F127 gel as the control group. F127 is composed of block copolymer of PEG and poly(propyl glycol) (PPG). The concentrated aqueous solution of F127 underwent a sol-gel transition upon heating as well. The Lira-loaded F127 gel exhibited a rapid release profile and almost all Lira was released within 2 days. It is obvious that the PCGA-PEG-PCGA thermogel is the optimal carrier for long-acting delivery of Lira among the materials examined in this study.

The release data of Lira from the three gel matrix were fitted via zero-order equation *Q* = *kt* and Higuchi equation *Q* = *kt*^1/2^, where *Q* is the cumulative release amount, *t* is release time, *k* is a constant. The fitted results are shown in Supplementary Table S2. As is well-known, Higuchi equation (*Q* < 0.6) represents a diffusion-controlled mechanism^41^. The release data of Lira from PCGA-PEG-PCGA and PLGA-PEG-PLGA thermogels well matched with Higuchi equation with the squared correlation coefficient *R*^2^ > 0.97, indicating that the diffusion-controlled mechanism governed the release of drug from the PEG/polyester copolymer hydrogels. For Pluronic F127, a nearly zero-order release was observed, which demonstrated that another release mechanism controlled the Lira release from F127 gel. In the section of Discussion, we will further explain the reasons of the different release mechanisms for these gel formulations.

### *In vivo* imaging of Lira from PCGA-PEG-PCGA thermogel

**Figure 5:**
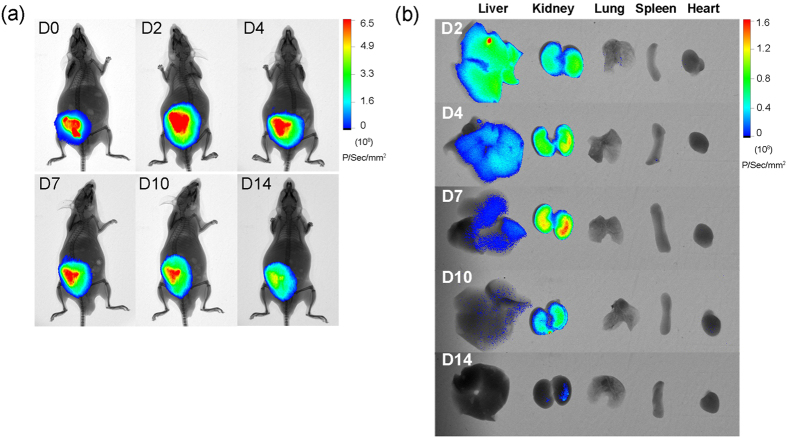
Monitoring of fluorescence-probe-modified peptide Cy5.5-Lira released from PCGA-PEG-PCGA thermogel. **(a)** The fluorescence of Cy5.5-Lira in ICR mice was monitored by non-invasive live imaging at different time points after SC injection. **(b)** *Ex vivo* organ distributions of degradation segments of Cy5.5-Lira at indicated days after SC injection. The livers, kidneys, lungs, spleens and hearts were isolated from mice at predetermined time points and fluorescence intensities were monitored. “D” denotes “day”.

Non-invasive imaging was further carried out to track the release of Lira from thermogel matrix in ICR mice. Considering that the Lira-loaded PCGA-PEG-PCGA gel formulation exhibited a sustained release profile *in vitro*, we choose it as the optimal formulation for the *in vivo* examination. The drug was fluorescently labeled by Cyanine 5.5 (Cy5.5). A strong NIR fluorescence was observed after SC injection of the thermogel formulation containing Cy5.5-Lira at the backs of ICR mice, as shown in Fig. 5a. The fluorescence was spread to the whole back of mice on day 2, and the core red fluorescence at the injection sites lasted for more than 10 days post-injection. Cy5.5 is a hydrophobic fluorescent probe with MW of 619. The introduction of Cy5.5 significantly increased the total hydrophobicity and MW of Lira, and thus strengthened the hydrophobic interaction and aggregation between polypeptide and carrier polymer, which resulted in a slower release rate from the thermogel depot.

Meanwhile, the major organs of mice were isolated and the fluorescence was observed in livers and kidneys. The fluorescence intensity in both the organs gradually decreased over timescale and almost disappeared on day 14 (Fig. 5b). It is well-known that Lira is metabolized by degradation within the body in multiple organs and tissues after SC administration, and the widely distributed endogenous enzymes, such as DPP-IV, are involved in the degradation of Lira^5^. Therefore, the fluorescence observed in livers and kidneys is attributed to the degradation segments of released Cy5.5-Lira, and they would be further eliminated from the circulation by glomerular filtration.

### *In vivo* hypoglycemic efficacy

**Figure 6:**
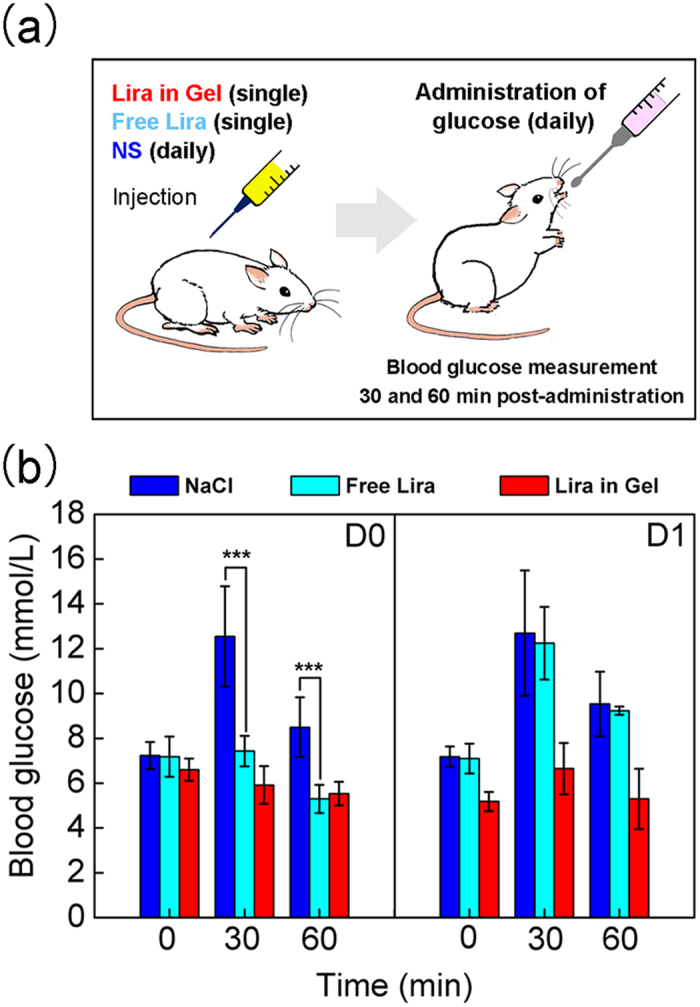
OGTT of ICR mice. **(a)** Schematic illustration of the procedure of the OGTT. To mimic the postprandial state, the mice were poured by glucose (3 g/10 mL/kg) once daily. Blood samples were collected at *t* = 0, 30 and 60 min for glucose measurements before and after the oral gavage of glucose. The data of blood glucose at *t* = 0 reflects the limosis blood glucose level before the oral gavage of glucose, while the data of blood glucose at *t* = 30 and 60 min after the oral gavage of glucose indicate the postprandial blood glucose levels. **(b)** Blood glucose levels in ICR mice in the groups “NaCl” (injection of 0.9% NS daily) “Free Lira” (a single injection of Lira solution) and “Lira in Gel” (a single injection of the Lira-loaded PCGA-PEG-PCGA gel formulation), *n* = 6 for each group. Here, the normal coordinate indicates the time after oral administration of glucose. “D0” in the legend denotes the day of injection, “D1” indicates the next day after the treatment. The significant differences between the group of “NaCl” and that of “Free Lira” are specifically marked: ***p < 0.001.

Compared with fasting glucose level, postprandial hyperglycemia is generally considered as a more sensitive indicator of diabetic control. One of widely used models to mimic the postprandial state of hyperglycemia is the oral glucose tolerance test (OGTT), which can rapidly reflect the hypoglycemic effect due to drug action on the glucose utilization^41^,^56^. In this study, we carried out OGTTs in ICR mice to evaluate the *in vivo* efficacy of the Lira-loaded PCGA-PEG-PCGA gel formulation on reduction of the blood glucose level at the postprandial state, as schematically presented in Fig. 6a.

The blood glucose levels of mice in the NaCl group rapidly increased after the oral administration of glucose. In contrast, the blood glucose levels of mice received a single SC injection of free Lira or Lira-loaded gel formulation significantly reduced at the time points of 30 and 60 min after the same administration of glucose on D0 (Fig. 6b). Unlike the long-acting Lira-loaded gel formulation, no sign of the hypoglycemic effect was observed for the group of Free Lira after oral gavage of glucose on the next day (D1). This finding coincides with the *in vivo* half-life of Lira^1^,^2^, suggesting that the repeated administration is required for the solution injection of Lira and thus the long-acting thermogel formulation is quite meaningful for the treatment of T2DM.

**Figure 7:**
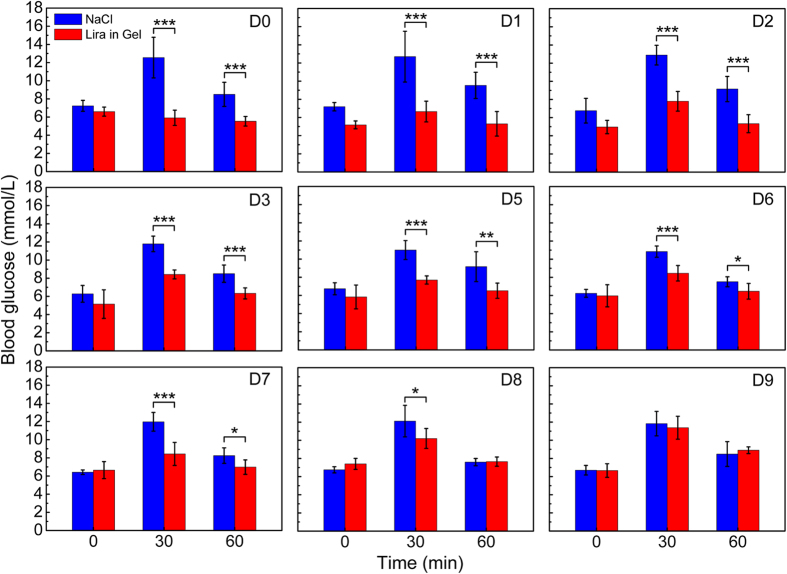
Blood glucose levels of ICR mice in the groups of “NaCl” and “Lira in Gel” during the whole experimental period of 10 days. *n* = 6 for each group. Here, the oral gavage of glucose in mice was once-daily performed, and the time points 0, 30, 60 min in the horizon coordination denotes the time after oral administration of glucose. “D0” in the legend denotes the day of injecting the Lira gel formulation, “D9” in the legend indicates day 9 after the treatment with the Lira gel formulation. In the group of “NaCl”, the physiological saline solution was injected daily. The hydrogel formulation of Lira was administrated via a single subcutaneous injection during the whole 10 days of *in vivo* examination. The continuous liberation of Lira from the hydrogel acted significantly the hypoglycemic efficacy within the initial 7 days. Significant differences between the group of “NaCl” and that of “Lira in Gel” are specifically marked: *p < 0.05, **p < 0.01, ***p < 0.001.

Subsequently, the OGTTs were carried out once per day during the whole experimental period of 10 days. The results in Fig. 7 demonstrated that there were significantly statistical differences of glucose level between the group of “NaCl” and that of “Lira in Gel” from D0 to D7 at the time points of 30 and 60 min after the administration of glucose. The *in vivo* tests indicated that the gel formulation of Lira had a significantly hypoglycemic effect up to one week in ICR mice after a single SC administration. This feature is well consistent with the *in vitro* release profile. Meanwhile, the blood glucose levels of mice that didn’t receive any treatment throughout the whole experiment period (as the group of “Blank”) were also detected as an indicator of the normal glucose level in the mouse blood. Their blood glucose levels were steady and no significant change was observed as a function of administration time, as demonstrated in Supplementary Fig. S2.

**Figure 8:**
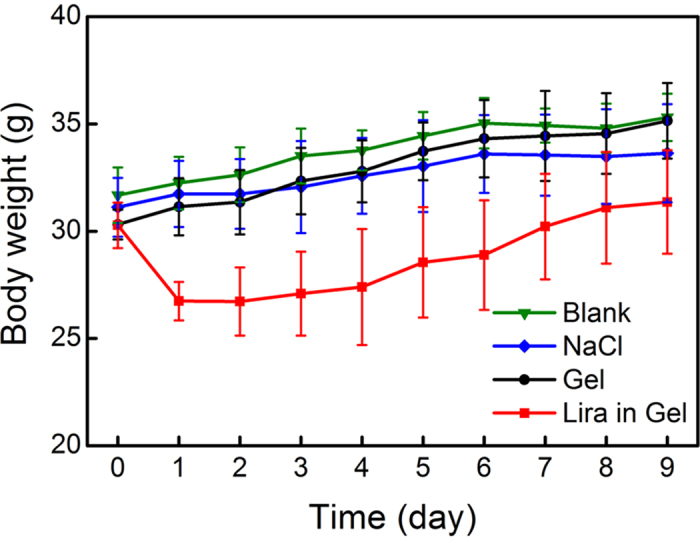
Body weights of ICR mice after the indicated treatments. *n* = 6 for each group. The lines are used just for the guides of the eyes.

Patients with T2DM are highly probably accompanied with obesity. Thus, how about the weight control of animals in the case of the Lira release? The mice were weighed daily, and the results are shown in Fig. 8. We set three control groups, the NaCl group, Gel group (without drugs) and Blank group (without any treatment). The body weight gradually increased over time in all of these control groups, and no statistic difference was observed among them. On the other hand, it has been widely recognized that the reduction of body weight is an index of *in vivo* toxicity of an implanted biomaterial. The change in body weight of mice received the administration of thermogel without any drug was similar to that of the Blank group, demonstrating the good biocompatibility of the thermogel itself.

In contrast, the body weight gain of mice in the group of Lira in Gel was significantly suppressed after the administration of Lira gel formulation. The mean body weight of mice received the Lira gel formulation was about 10% lower than that of the blank. This feature affirmed that the released Lira has the potency of delaying gastric emptying and reducing appetite, which is in accordance with the pharmacology of Lira as illustrated elsewhere^8^,^9^.

## Discussion

It is well-recognized that the development of new T2DM treatments should avoid the decline of *β*-cell function, occurrence of hypoglycemia and body weight gain. Lira is a “smart” glucoregulatory agent associated with *β*-cell function improvement, glucose-dependent insulin secretion and body weight reduction, and it exerts the hypoglycemic efficacy only when blood glucose levels are higher than normal^5^,^6^. Namely, it shows negligible risk of hypoglycemia in spite of the sustained release of Lira. Nevertheless, the solely commercial formulation of Lira is its solution injection and daily injection is still not ideal for the treatment of T2DM. Therefore, the development of long-acting delivery system of Lira is much desired.

Injectable thermogels composed of PEG and biodegradable polyesters have been suggested as potential carriers for sustained delivery of various drugs, such as protein, polypeptide and small-molecule drug^33^,^42^,^49^,^57^,^58^,^59^. However, there is little work on release kinetics comparisons between carrier polymers with different polyester components for the same model drug. In the present study, two triblock copolymers PCGA-PEG-PCGA and PLGA-PEG-PLGA with similar MWs were synthesized by us. Both the polymer aqueous solutions underwent reversible sol-gel transitions as the temperature increased and formed semi-solid thermogels at body temperature (Fig. 2). Hence, the two thermogels were selected as the carriers to deliver Lira for the first time, and their release kinetics comparisons were carried out as well.

It is well-known that the PEG/polyester copolymers could form core-corona micelles in water with the hydrophilic PEG blocks locating in the coronas and the hydrophobic polyester blocks in the cores^36^,^39^. Herein, the formation of PEG/polyester micelles was also confirmed by DLS measurements, and the size of copolymer micelles slightly increased with addition of Lira (Fig. 3a). Also, the changes in the secondary structure of Lira were observed by CD analysis (Fig. 3b). On a macro level, the introduction of Lira resulted in a significant increase in *η* of thermogels (Fig. 3c). Taken together, we believe that there were the interactions between Lira and the carrier polymers. Considering that Lira is an amphiphilic polypeptide with a hydrophobic 16-carbon side chain, we further speculate that the C16 side chain probably entered the hydrophobic cores of micelles through the hydrophobic interaction with each other.

Non-degradable F127 hydrogel is easily eroded in water, resulting in its fast disappearance within one or two days^34^. As a result, Lira was thoroughly released from the F127 gel matrix due to the rapid erosion of carrier polymers (Fig. 4). In contrast, the PEG/polyester thermogels can maintain their *in vivo* integrities from one week to several months, and their degradation mainly depends on the hydrolysis of polyester segments and the final degradation products are lactic acid, glycolic acid, 1,6-hydroxycaproic acid and PEG, which are nontoxic and easily cleared from the body^12^,^33^,^34^,^46^,^57^. Consequently, the PEG/polyester thermogels are suitable for long-acting drug delivery. Nevertheless, the hydrophilic polypeptides, such as insulin and exenatide, are often enclosed between micelles after being loaded into the thermogel depots, and thus their release period is only a few days because of the fast diffusion of drugs^41^,^59^. In this study, although the release of Lira, an amphiphilic polypeptide, from the thermogels was also governed via diffusion mechanism, a sustained release manner without significant initial burst was observed for the two gel formulations. This feature was attributed to the hydrophobic interactions between C16 fatty side chain of Lira and the hydrophobic cores of copolymer micelles, which effectively delayed the diffusion of drug. And yet, due to the difference of polyester components, the two thermogel systems also exhibited the different diffusion rates of drug.

Now, a question arises: what kind of intrinsic factor leads to the different release profiles? Compared with PLGA segment, the PCGA block was more hydrophobic because of the longer hydrocarbon chain in the PCL backbone^54^. However, the diffusion of Lira from the PCGA-PEG-PCGA thermogel system was faster even though the hydrophobic interaction between drug and the carrier polymer seemed to be relatively stronger. Hence, the difference of hydrophobic and hydrophilic performance of polyester components was not the intrinsic factor. Over the past decades, PLGA, PCL and their copolymers have been widely utilized as carrier polymers for drug delivery in different forms including microsphere, nanoparticle, micelle and hydrogel^60^,^61^,^62^,^63^. Interestingly, a very similar trend had been reported, namely, the release rate of drug from PCL-based systems was often faster than that of PLGA systems^51^,^52^,^60^,^61^,^63^,^64^. The highly flexible nature of PCL chain, which produced the high mobility of chain and the good permeability to drugs, was accounted for the increased release rate. In the present study, a much lower *T*_g_ of PCGA-PEG-PCGA when compared to PLGA-PEG-PLGA polymer also reflects the highly flexible nature of PCGA block. Meanwhile, this result suggests that the PCGA-PEG-PCGA system has the higher chain motion at body temperature compared with PLGA-PEG-PLGA, and thus facilitates the diffusion of drug. As a result, the PCGA-PEG-PCGA/Lira system exhibited a sustained release profile *in vitro* over one week. The *in vivo* imaging also demonstrated that this gel formulation exhibited a long-acting drug release manner in an animal model (Fig. 5). In contrast, in the case of PLGA-PEG-PLGA/Lira system, the low mobility of carrier polymer resulted in an incomplete release of Lira at the late stage due to the difficulty of drug diffusion out of the micelles.

**Figure 9:**
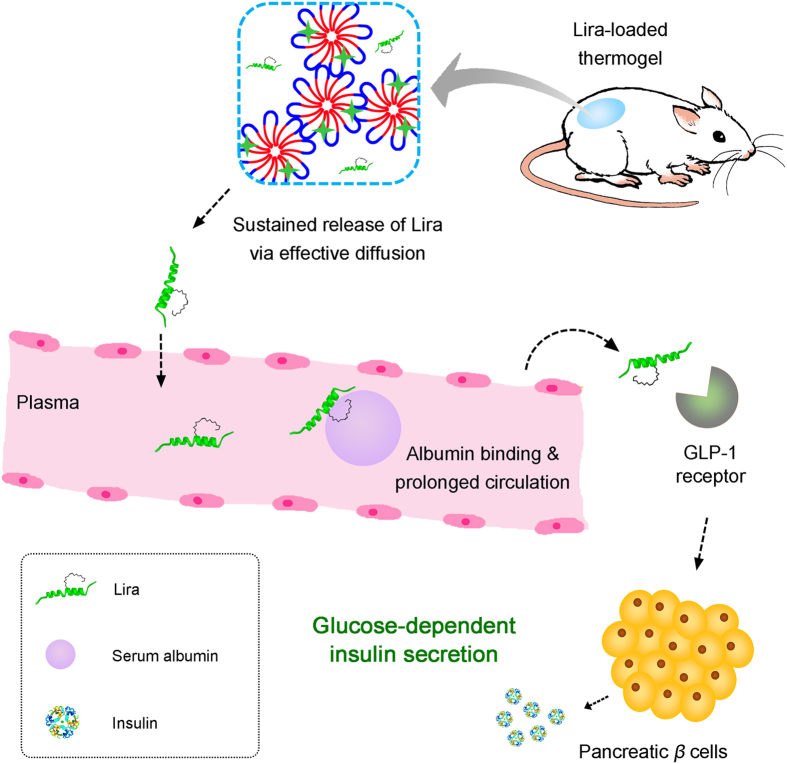
Schematic presentation of controlling blood glucose level by the Lira-loaded PCGA-PEG-PCGA thermogel formulation. The copolymer aqueous system became a physical hydrogel at body temperature after injection due to the formation of a percolated micelle network upon heating. The combination of an appropriate drug/polymer interaction and a high chain mobility of carrier polymers led to a sustained release of Lira from the thermogel matrix. The circulation of Lira was remarkably prolonged via its non-covalent binding to serum albumin. The activation of GLP-1 receptors on pancreatic *β* cells by Lira promoted insulin secretion in a glucose-dependent manner, leading to intelligent blood glucose reduction.

After a single SC administration of Lira-loaded PCGA-PEG-PCGA gel formulation in ICR mice, the hypoglycemic effect lasted up to one week (Fig. 7) and such a feature coincided with the *in vitro* release profile. Figure 9 schematically presents the mechanism of the thermogel formulation in controlling blood glucose level. The successful animal testing also affirmed the bioactivity of drug released from the gel depot. This feature indicates that the denaturation of Lira in the gel matrix did not occur even if the secondary conformation of Lira changed due to its interaction with carrier polymers. Moreover, the administration of Lira gel formulation induced modest weight loss (Fig. 8), which is beneficial for some patients of T2DM with obesity. In fact, the Lira solution injection Saxenda (Novo Nordisk Inc.) has been approved by FDA in 2014 as a treatment option for chronic weight management. Hence, besides as a sustained release anti-diabetic system, our Lira-loaded thermogel formulation may also serve as a long-acting weight-manage formulation for patients who are obese or overweight.

## Conclusion

This paper reports the first long-acting drug delivery system of Lira, a glucose-dependent insulinotropic hormone. To this end, injectable and biodegradable PCGA-PEG-PCGA and PLGA-PEG-PLGA thermogels were synthesized. Both Lira and the PEG/polyester polymers were amphiphilic, and their mixtures easily formed micelles in water. The significantly different release profiles were observed in the two Lira-loaded thermogel systems with varying polyester components. As one of the two cases, the sustained release of Lira from the PCGA-PEG-PCGA gel formulation with a period of one week was achieved. This result was attributed to both the appropriate drug/carrier polymer interaction and the high chain mobility of carrier polymer. Their combination led to the effective diffusion of drug from the hydrogel matrix for a relative long time.

After a single shot of the Lira-loaded PCGA-PEG-PCGA gel formulation into mice, not only an *in vivo* sustained release was observed, but also a reduced blood glucose level at the postprandial state was achieved up to one week. The *in vivo* therapeutic efficacy was consistent with the *in vitro* release profile. Consequently, the PCGA-PEG-PCGA thermogel holds great promise for once-weekly delivery of Lira, and such a delivery system can improve patient compliance significantly. Meanwhile, the strategy based on the interactions between drugs and carrier polymers and the chain mobility of carrier polymers to control drug release might be helpful for the development of some other kinds of drug delivery systems.

## Methods

### Materials

PEG of MW 1500, *ε*-caprolactone (CL), Pluronic F127, and stannous 2-ethyl-hexanoate (stannous octoate, 95%) were supplied by Sigma-Aldrich (USA). Glycolide (GA) and D,L-lactide (LA) were purchased from Purac (Netherlands). Liraglutide was synthesized by Chinese Peptide Co., Ltd (Hangzhou, China). Cyanine5.5 N-hydroxysuccinimide ester (Cy5.5-NHS) was product of Lumiprobe (USA). All of chemicals were used without further purification.

### Experimental animals

ICR mice (male, 30 ± 2 g) were purchased from SLAC Laboratory Animal Co. Ltd (Shanghai, China). The animals were housed in cages at 22~25 °C with 12–12 h light-dark cycle. During acclimation, the mice were fed with a standard laboratory chow diet and tap water. All the animal experiments were carried out in accordance with the approved guidelines of the “Principles of Laboratory Animal Care” (NIH publication #85-23, revised 1985) and were approved by the ethics evaluation board of Fudan University.

### Synthesis and physicochemical characterization of polymers

Triblock copolymers of PCGA-PEG-PCGA and PLGA-PEG-PLGA were synthesized via ring-opening polymerization of CL and GA or LA and GA using PEG as the macroinitiator and stannous octoate as the catalyst. The detailed synthetic procedure has been described in our previous publications^28^,^35^. ^1^H NMR was used to determine the composition of triblock copolymers. Spectra were recorded at a 500 MHz Bruker spectrometer (DMX500) using CDCl_3_ as the solvent. MWs of copolymers and the distribution thereof were measured using a gel permeation chromatography system (GPC, Agilent 1100). The eluent was tetrahydrofuran at a flow rate of 1.0 mL/min at 35 °C. Uniform polystyrene standards were used to obtain a calibration curve. The *T*_g_ of copolymers was determined by DSC (Q2000, TA Instruments) in the temperature range from −80 °C to 80 °C with a heating rate of 20 °C/min. The sample was loaded in an aluminum pan and scanned in the instrument to record the thermograms. The midpoint of the transition zone was defined as *T*_g_.

The vial inverting method^25^,^41^ with an increment temperature of 1 °C per step was used to determine the sol-gel transition of the polymer aqueous solution. Rheological characteristics of the polymer aqueous solutions with or without drug was also measured using a stress-controlled rheometry (Kinexus Pro, Malvern) equipped with a Peltier plate (1° steel cone, 60 mm diameter with solvent trap)^47^. The time sweep measurements were carried out at a heating rate of 0.5 °C/min and an oscillatory frequency of 10 rad/s.

### Dynamic laser scattering

The hydrodynamic diameters of copolymer micelles in PBS with or without Lira were measured by dynamic light scattering instrument (Zetasizers Nano ZS90, Malvern) with a vertically polarized incident beam at 532 nm and a fixed scattering angle of 90°. All of the samples were filtered through 0.45-μm filters and equilibrated at room temperature for 12 h before measurement.

### Circular dichroism spectroscopy

The secondary structures of Lira with or without copolymers were characterized by circular dichroism instrument (Bio-Logic MOS-450, Bio-Logic). Spectra were recorded from 200 to 280 nm using a bandwidth of 1 nm and a scanning rate of 1 nm/s. Aqueous polymer solutions without Lira were also scanned in the same wavelength range as the blank background.

### *In vitro* drug release

Lira was added into polymeric aqueous solutions (PBS, pH 7.4, polymer concentration: 25 wt%) and stirred at 4 °C till it was completely dissolved. Then, 0.5 mL polymer solutions containing 2 mg/mL Lira were transferred into 10-mL test tubes (inner diameter 14 mm), and the samples were converted to physical hydrogels by incubating in water bath at 37 °C for 10 min. Next, 5 mL of PBS (pH 7.4, 37 °C) containing 0.025% NaN_3_ was added as release medium. The shaking rate of water bath was fixed at 50 rpm. At designated time intervals, the release medium was totally extracted from the tubes and replaced with the same amount of fresh buffer to maintain the sink condition. The drug amount released into the release medium were measured by high-performance liquid chromatography (HPLC, Waters Separation Module e2695) equipped with a C18 column (5 μm particle, 150 × 4.6 mm, Phenomenex). Mobile phase A was acetonitrile and mobile phase B was water containing 0.1% trifluoroacetic acid. A flow-rate of 1.0 mL/min was set over 20 min with mobile phase A increased from 48% to 56% and mobile phase B decreased from 52% to 44%. Detection was performed with a UV detector (Waters UV/visible detector 2489) at 220 nm.

### Monitoring of Lira release from thermogel in ICR mice

*In vivo* real-time fluorescence imaging was performed in ICR mice for monitoring of Lira release from thermogel. The polypeptide was labeled with a Cy5.5 dye by adding Cy5.5-NHS to Lira solution at pH 8.4 with a molar ratio of 1/1. After incubated at 4 °C for 12 h, the solution was dialyzed to remove the unreacted Cy5.5 and then freeze-dried. PCGA-PEG-PCGA solutions (25 wt%, 0.2 mL) containing Cy5.5-Lira were SC injected into the backs of ICR mice. At predetermined time points, the fluorescence of the whole body and isolated organs including liver, kidney, lung, spleen and heart were monitored by a small animal imaging system (*In-Vivo* Xtreme, Bruker) with an excitation wavelength of 690 nm and emission wavelength of 790 nm.

### Mice oral glucose tolerance test

Mice OGTTs^41^,^56^ were performed to evaluate the *in vivo* hypoglycemic efficacy of the Lira-loaded PCGA-PEG-PCGA thermogel formulation at the postprandial state. The experimental mice were randomly divided into 5 groups with 6 mice per group. For the NaCl group, the mice received a SC injection of 0.9% NaCl solution (NS) daily at a dosage of 7.5 mL/kg. For the Gel group, the mice received a single SC injection of thermogel without drugs. The group of Free Lira was administrated by a single SC injection of Lira solution at a dosage of 1.5 mg/7.5 mL/kg. The group of Lira in Gel was administrated by a single SC injection of Lira-loaded gel formulation (15 mg/7.5 mL/kg). For the Blank group, the mice didn’t receive any treatment throughout the whole experiment period. The group of Free Lira was only applied for the short-time examination, while the group of Lira in Gel as a long-acting drug delivery system was evaluated in both the short-time (1 day) and long period (10 days), and thus the drug dosage in the group of Lira in Gel was 10-fold of that of the Free Lira group. All of the mice were fasted from 9:00 a.m. for 4 h but allowed to drink water before treatment. For mice of NaCl group, Free Lira group, Lira in Gel group and Blank group, about 30 μL basal blood sample was obtained from the tail vein (defined as *t* = 0) to measure the glucose concentration using a one-touch blood glucose monitoring system (Accu Check Active, Roche Diagnostics). The mice in the groups of NaCl, Free Lira and Lira in Gel were poured by glucose (3 g/10 mL/kg) after different injections for 15 min. Then, blood samples were collected at *t* = 30 and 60 min for glucose measurements after the oral gavage of glucose.

On the next 9 days post-administration, the mice were fasted from 9:00 a.m. for 4 h. Subsequently, blood samples were taken from the tail vein (*t* = 0) to determine baseline glucose levels before oral administration of glucose (the same as above). And at *t* = 30 and 60 min after oral administration of glucose, blood samples were also collected and detected. Meanwhile, body weights of mice were recorded each day before fasting.

### Statistical analysis

All the results were expressed as mean ± standard deviation (SD). For group comparison, the statistical differences of mean values were analyzed by least significant difference (LSD) *t*-tests. A *p* value of <0.05 was considered as a significant difference.

## Additional Information

**How to cite this article**: Chen, Y. *et al.* Controlled release of liraglutide using thermogelling polymers in treatment of diabetes. *Sci. Rep.* **6**, 31593; doi: 10.1038/srep31593 (2016).

## Supplementary Material

Supplementary Information

## Figures and Tables

**Figure 1:**
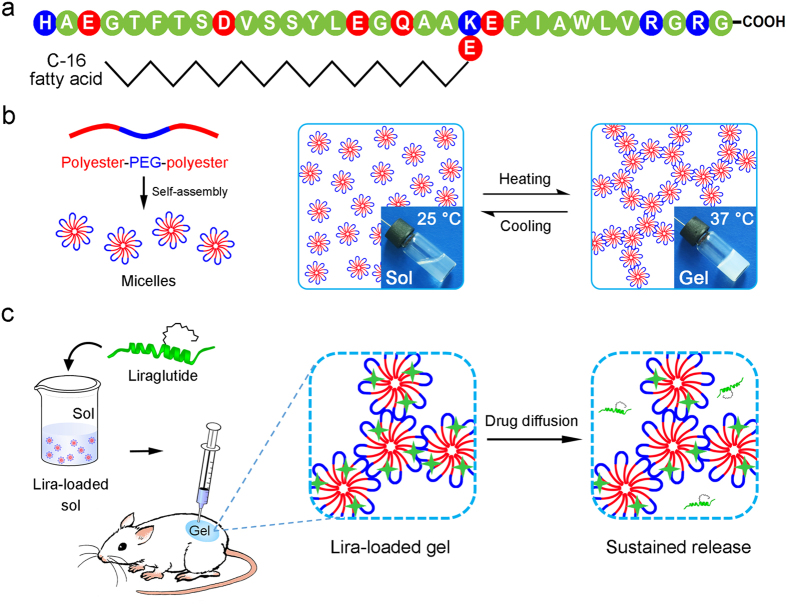
Schematic presentation of a Lira-loaded thermogel formulation as the long-acting drug delivery system. **(a)** Amino acid sequence of Lira with neutral residues in green, acidic ones in red and alkaline ones in blue. **(b)** Amphiphilic polyester-PEG-polyester triblock copolymers self-assemble into micelles in water at room temperature and turn into a physical hydrogel due to the formation of a percolated micelle network at body temperature. **(c)** Main procedures of constructing the Lira-loaded thermogel formulation, its *in vivo* application and the corresponding drug release mechanism.
