# Supplementary material for: Controlled release of liraglutide using thermogelling polymers in treatment of diabetes
Source: Sci Rep. 2016 Aug 17;6:31593. doi: 10.1038/srep31593 (PMC4987673; doi:10.1038/srep31593)
Supplement: Supplementary Information [file srep31593-s1.pdf]

SUPPLEMENTARY INFORMATION

Controlled release of liraglutide using thermogelling polymers in treatment of diabetes

Yipei Chen[1], Yuzhuo Li[1], Wenjia Shen[1], Kun Li[2], Lin Yu[1,*], Qinghua Chen[2] & Jiandong Ding[1]

[1] State Key Laboratory of Molecular Engineering of Polymers, Collaborative Innovation Center of Polymers and Polymer Composite Materials, Department of Macromolecular Science, Fudan University, Shanghai, 200433, China

[2] National Pharmaceutical Engineering Research Center, China State Institute of Pharmaceutical Industry, Shanghai, 200437, China

* Correspondence and requests for materials should be addressed to L.Y.

(email: yu_lin@fudan.edu.cn)

Supplementary Figures

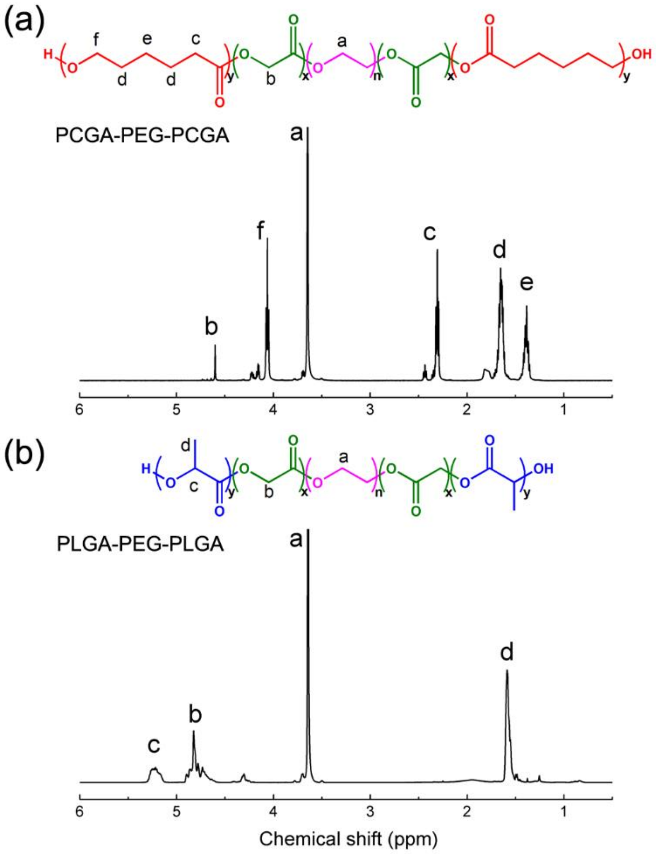

Figure S1. ^{1}H NMR spectra of the synthesized triblock copolymers in $CDCl_3$. (a) PCGA-PEG-PCGA, (b) PLGA-PEG-PLGA.

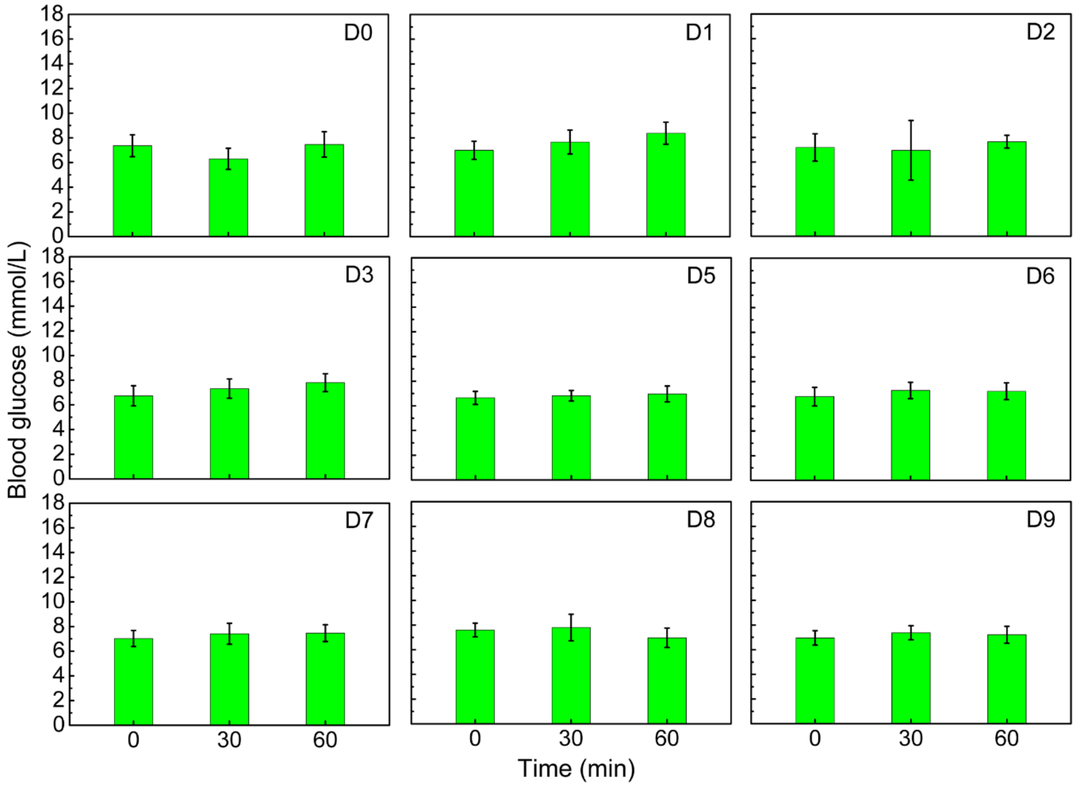

Figure S2. Blood glucose levels in ICR mice in the groups of "Blank", *n* = 6 for each group. The mice didn't receive any treatment throughout the whole experiment period. The blood glucose levels were detected as an indicator of the normal glucose level. No significant change was observed as a function of administration time. Here, the time points 0, 30, 60 min in the horizon coordination denote the time after oral administration of glucose in mice of group "NaCl" and "Lira in Gel". "D0" in the legend denotes the day of injecting the Lira gel formulation, "D9" in the legend indicates day 9 after the treatment with the Lira gel formulation.

Supplementary Tables

Table S1. Parameters of the triblock copolymers synthesized in this study

Specimen	M_n[a]	Molar ratio (mol/mol)[a]	M_n[b]	(M_w/M_n)[b]	T_g[c]
PCGA-PEG-PCGA	1825-1500-1825	CL/GA 90/10	7530	1.33	-57 ℃
PLGA-PEG-PLGA	1780-1500-1780	LA/GA 50/50	6650	1.21	-4 ℃

[a)] The M_n of the central block PEG was provided by Aldrich. The molar ratios of repeating units and M_n of each polyester block were calculated by ^{1}H-NMR;

[b)] Measured by GPC, relative to polystyrene standards.

[c)] Measured by DSC.

Table S2. Kinetic assessments of the *in vitro* release data from the different gel matrix

Sample	$Q = kt$		$Q = kt^{1/2}$	
	k	R^2	k	R^2
PCGA-PEG-PCGA	0.120	0.945	0.311	0.978
PLGA-PEG-PLGA	0.077	0.958	0.197	0.998
Pluronic F127	0.456	0.975	0.347	0.836
